# The Extended Safety and Efficacy of Indobufen in Conjunction With P2Y12 Receptor Inhibitors Among Patients Undergoing Revascularization: A Meta-Analysis and Overview

**DOI:** 10.1155/cdr/5374818

**Published:** 2025-11-24

**Authors:** Ming Yi, Qing Cao, Yang-hui Sheng, Lu Wu, Xiao Ke

**Affiliations:** ^1^Department of Cardiology, Liuyang Hospital of Traditional Chinese Medicine, Liuyang, China; ^2^Department of Cardiology, Fuwai Hospital, Chinese Academy of Medical Sciences, Shenzhen, (Shenzhen Sun Yat-sen Cardiovascular Hospital), Shenzhen, China; ^3^Department of Cardiology, The Fourth Hospital of Changsha, Changsha Hospital Affiliated to Hunan Normal University, Changsha, China

**Keywords:** coronary artery disease, indobufen, P2Y12 receptor inhibitors, revascularization

## Abstract

**Background/Objectives:**

Dual antiplatelet therapy (DAPT), which combines aspirin with a P2Y12 receptor inhibitor for platelets, is crucial for the prevention of cardiac and systemic ischemic events in patients with coronary artery disease who have undergone revascularization procedures. Although indobufen is suggested as an alternative for individuals who exhibit intolerance to aspirin, the long-term safety and efficacy of indobufen-based DAPT treatment for the prevention of cardiac and systemic ischemic events in these patients remain ambiguous. This meta-analysis seeks to examine the long-term safety and efficacy of DAPT based on indobufen in the context of revascularization procedures for patients.

**Methods:**

A thorough search was performed across PubMed, Embase, the Cochrane Library, Web of Science, ClinicalTrials.gov, and CNKI, covering all records from each database's inception to October 10, 2024. Included were randomized trials analyzing oral DAPT antiplatelet agents for coronary artery disease patients who received revascularization. Two reviewers independently handled the selection process: screening articles, overview, extracting data, and assessing study quality in accordance with PRISMA guidelines. The pooled data were later subjected to analysis using a random-effects model meta-analysis.

**Results:**

The final analysis included three randomized controlled trials, which yielded the following findings regarding major adverse cardiovascular and cerebrovascular events (MACCEs): The relative risk (RR) was 1.58 (95% CI, 0.72–3.38); for BARC Type 2, 3, or 5 bleeding events, the RR was 0.35 (95% CI, 0.18–0.67); and for gastrointestinal intolerance events, the RR was 0.06 (95% CI, 0.03–0.18).

**Conclusions:**

Indobufen-based DAPT may potentially increase the risk of ischemic events without compromising safety for revascularization, as its upper RR limit exceeds 1 for MACCEs.

## 1. Introduction

Coronary atherosclerotic heart disease (CAD) is becoming increasingly common, making it a significant contributor to mortality rates [1]. In the development of CAD, both arterial and venous thrombosis play a prevalent role. Antiplatelet therapy plays a crucial role in CAD treatment due to the essential involvement of platelet activation and aggregation in atherothrombotic formation. Excessive platelet activation contributes to thrombus formation, which may lead to severe cardiovascular events such as myocardial infarction. Therefore, antiplatelet drugs like aspirin and clopidogrel are widely used in the prevention and treatment of CAD to reduce thrombotic risks. These drugs safeguard cardiac health in patients by blocking platelet aggregation. Notably, their efficacy is particularly evident in scenarios such as acute myocardial ischemia syndrome, postpercutaneous coronary intervention (PCI), coronary artery bypass grafting (CABG), and long-term secondary prevention strategies. However, balancing antithrombotic benefits with bleeding risks remains a clinical challenge, necessitating personalized risk assessment and treatment adjustment [2]. Platelet cyclooxygenase (COX)-1 inhibitors, mainly aspirin and indobufen, are the most commonly used antiplatelet agents [3]. Aspirin inhibits platelet aggregation by irreversibly inhibiting COX-1, thereby blocking the synthesis and release of thromboxane A2 [4]. Indobufen, a selective and reversible inhibitor of the COX-1 enzyme, exerts its action by impeding the synthesis of proinflammatory prostaglandins. This mechanism of action renders it less likely to provoke adverse gastrointestinal reactions and hemorrhagic events, in contrast to other nonsteroidal anti-inflammatory drugs (NSAIDs) that extensively block both COX-1 and COX-2 enzymes. This lower risk profile is attributable to indobufen's more precise targeting of COX-1, which is primarily responsible for maintaining the integrity of the gastrointestinal mucosa, without unduly affecting the COX-2 enzyme that plays a pivotal role in inflammatory processes. As a result, indobufen emerges as a relatively safer therapeutic option for patients who require long-term antiplatelet therapy or pain management, minimizing the potential for severe gastrointestinal complications that can often accompany the use of more potent, nonselective NSAIDs [3–5]. Earlier studies have shown that indobufen is significantly more effective than a placebo in secondary prevention. Numerous clinical trials have demonstrated that indobufen can substantially lower the occurrence of myocardial and cerebrovascular ischemia, and it is well tolerated in individual patients. These research results provided a solid foundation for further exploration and application of indobufen and also aroused wide attention in the medical field [6].

Earlier meta-analyses have assessed indobufen's effectiveness for preventing thromboembolic complications, including in patients with atrial fibrillation, as well as for preventing graft occlusion following CABG and in-stent restenosis after procedures involving stenting [7–9]. Current research indicates that indobufen may serve as a suitable alternative to aspirin in addressing cerebrovascular, peripheral vascular, and coronary vascular diseases while exhibiting a reduced occurrence of gastrointestinal complications, making it more suitable for long-term therapy [5, 10, 11].

A recent meta-analysis conducted an evaluation of the safety and efficacy of indobufen in the treatment of cardiovascular, cerebrovascular, and thromboembolic diseases [12]. The primary focus of this study was the incidence of major adverse cardiovascular events (MACEs), thrombosis, bleeding events, and other adverse occurrences. The findings concluded that indobufen was associated with a reduced risk of adverse events and bleeding complications [12]. This meta-analysis included a total of 12 studies; however, the conclusions drawn are subject to further discussion and analysis. Firstly, the time span of the literature encompassed is extensive. Given the rapid advancements in vascular intervention technology, the benefits of revascularization have significantly improved, which may not have been fully assessed in earlier studies. Secondly, indobufen was not adequately compared against a control group. Thirdly, it is not possible to eliminate the disparities within the study populations. Finally, peripheral vascular disease was excluded from the analysis. The varying applications of induobufen merit further discussion and investigation.

Meta-analyses demonstrate that in patients undergoing complex PCI, P2Y12 inhibitor monotherapy provides enhanced safety in terms of major bleeding episodes and does not elevate the risk of ischemic events when compared to standard dual antiplatelet therapy (DAPT). Additionally, it appears to reduce the risk of myocardial infarction; however, this requires validation through targeted clinical trials [13]. Consequently, treatment guidelines consistently endorse the use of aspirin-based DAPT as the subsequent therapeutic approach in patients presenting with acute coronary syndrome (ACS) [13, 14].

However, some adverse effects of aspirin have limited its use in clinical practice [14, 15]. Intensive antiplatelet therapy is necessary for patients with chronic coronary syndrome (CCS) who undergo revascularization. The risk of bleeding remains a concern that must be addressed to ensure the optimal use of DAPT, maximizing the benefits of antithrombotic therapy while minimizing bleeding risks. Indobufen has been found to attenuate the adverse effects of aspirin while maintaining antithrombotic efficacy [11]. However, there is no systematic evaluation of indobufen in combination with P2Y12 receptor inhibitors.

This study seeks to assess the long-term safety and efficacy of an indobufen-based DAPT as a background treatment for patients with ACS and CCS undergoing revascularization. It will review prior meta-analyses and integrate findings from new randomized controlled trials (RCTs). The findings of this research are anticipated to offer supplemental references and substantial evidence to support its application in clinical practice. With the continuous rise in the incidence of ACS and CCS, it is of paramount importance to explore more efficient and secure therapeutic strategies to minimize patient risks and enhance their quality of life.

## 2. Methods

This overview and meta-analysis were conducted following the PRISMA statement and registered with INPLASY to ensure transparency and reproducibility. The PRISMA guidelines were meticulously adhered to, guaranteeing a thorough and comprehensive assessment of the literature (the flow diagram was generated using a Shiny App available at https://www.eshackathon.org/software/PRISMA2020.html). Furthermore, registration with INPLASY (https://inplasy.com) enhances the study's openness and replicability, allowing other researchers to better understand and validate the methods employed [16].

### 2.1. Methodology for Information Retrieval

Ke Xiao developed a thorough and comprehensive retrieval strategy for the study, with Yi Ming and Lu Wu independently conducting optical searches in the Web of Science, PubMed, Embase, and Cochrane databases to find studies published up until October 2024. Qing Cao and Yang-hui Sheng reviewed the data. The search utilized keywords such as “Indobufen,” “Yinduobufen,” “Ibostrin,” “Yinduo,” and “K-3920.” Subsequent to the preliminary search, the literature underwent a meticulous screening process, and references from the studies that were included were examined manually for any additional pertinent publications. The flow diagram illustrates the comprehensive literature search process.

### 2.2. Criteria for Considering Studies for This Review

This review was meticulously crafted to encompass solely RCTs and propensity score matching (PSM) studies, thereby guaranteeing that the results presented are grounded in the most stringent and objective research methodologies available. Patients diagnosed with CAD complicated by myocardial ischemia, who underwent revascularization and received oral DAPT agents, were recruited from these RCTs and PSM studies. The studies eligible for inclusion in the analysis are determined by the following criteria: (1) design: RCT/PSM, registered clinical trials, and disclosure of ethical details; (2) patients: all participants diagnosed with CAD; and (3) revascularization procedures, which encompass percutaneous coronary stent implantation, percutaneous coronary balloon dilatation, or CABG. The trial group received indobufen in conjunction with clopidogrel or ticagrelor, whereas the control group was administered aspirin in combination with clopidogrel or ticagrelor. Conversely, the following criteria led to the exclusion of studies: (1) the use of alternative efficacy evaluation indicators, (2) involvement of healthy participants in the control group, (3) insufficient data from the experimental sample, (4) instances of duplicate publication, (5) pharmacokinetic studies, (6) meta-analyses, and (7) failure to disclose randomization control details.

### 2.3. Defined Endpoints

The main efficacy endpoint was MACCE, which is a broad measure that includes various cardiovascular events. This composite endpoint included cardiovascular death; nonfatal myocardial infarction, which signifies damage or injury to the heart muscle without complete blockage of blood flow; clinically driven repeated revascularization, indicating the need for additional procedures to restore blood flow; definite or probable stent thrombosis, a potentially life-threatening condition where a blood clot forms in the stent; and nonfatal ischemic stroke, resulting from inadequate blood supply to the brain. The primary safety endpoint was the Bleeding Academic Research Consortium (BARC) criteria, a standardized classification system used to assess the severity and types of bleeding events during clinical trials.

### 2.4. Evaluation of Quality

The RevMan tool and R software were employed to assess the quality of the studies incorporated within the complete text articles. The evaluation centered on these criteria: (1) generation of random sequences, (2) concealment of allocation, (3) blinding of both participants and personnel, (4) outcome assessment blinding, (5) management of incomplete outcome data, (6) reporting selection, and (7) other possible bias sources.

### 2.5. Collecting Insights From Data

Qing Cao and Ming Yi independently gathered data from publications utilizing electronic files that adhered to established criteria. From each article, we extracted the following information: investigator, study year, design, participant count, average age, type of treatment, follow-up duration, risk ratio (RR), corresponding 95% confidence intervals, and specifics regarding cases in both the intervention and control groups. We reached out to the corresponding authors when univariate or multivariate hazard ratios (HR) were missing. Studies were excluded if we could not obtain additional information.

### 2.6. Statistical Analysis

The intervention and control group data were analyzed to evaluate binary outcomes. We assessed the heterogeneity in studies through the Cochrane nonparametric test (*Q*, *I*-square, StatsDirect). When the value of *I*^2^ exceeded 30% or *p* was less than 0.05, indicating substantial heterogeneity, sensitivity and subgroup analyses were conducted to assess each study's impact on the overall results. LogRR values computed by the SAS software, as presented in the main text for clarity and ease of understanding, have been converted so that all logRR calculations are expressed as RR values. A random-effects model yielded aggregated estimates. We evaluated the potential for publication bias using a funnel plot and Egger's test. Statistical analyses were performed using Stata software Version 16 (StataCorp LLC, 4905 Lakeway Drive, College Station, Texas), with *p* values deemed statistically significant if below 0.05.

## 3. Results

### 3.1. Studies Overview

**Figure 1:**
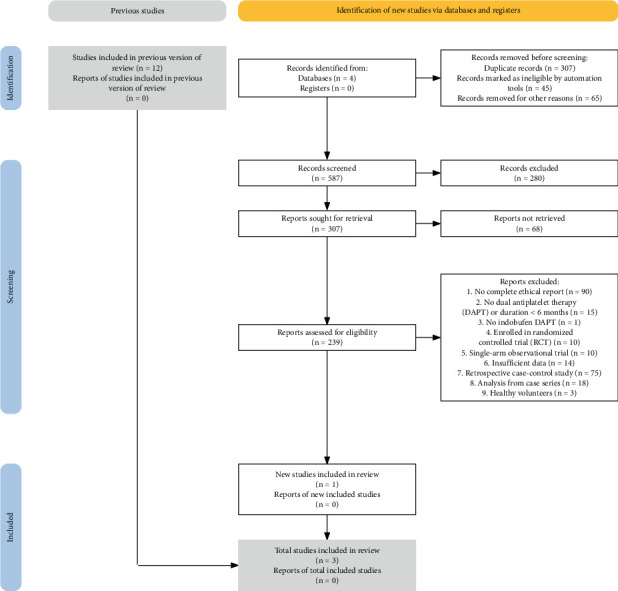
Flowchart depicting eligible studies for the meta-analysis.

In accordance with our designated retrieval strategy (refer to Figure 1), a previously published meta-analysis was reanalyzed, and a total of 587 records were identified following the elimination of duplicates. Subsequent to the screening of titles and abstracts, 220 articles were excluded from consideration. A comprehensive review of the full texts identified 31 articles, of which three articles [17–19] were ultimately incorporated in this overview and meta-analysis. The review included a total of 2556 patients who had undergone oral DAPT with indobufen, with a mean age of 61.7 ± 1.2 years.  Table 1 summarizes the characteristics of the patient study.

### 3.2. Primary Efficacy Endpoint

**Figure 2:**
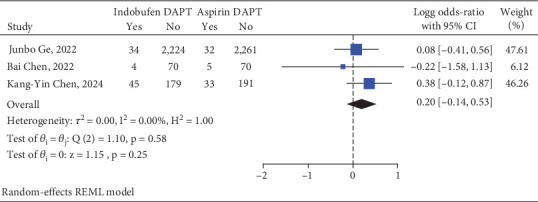
Indobufen-based DAPT versus aspirin-based DAPT on the primary efficacy endpoint.

The primary efficacy endpoints, which are categorized as adverse events, were documented in all three RCTs. The primary endpoint was observed in 83 patients (3.25%) within the indobufen-based DAPT group, while it occurred in 70 patients (2.70%) in the aspirin in combination with P2Y12 receptor antagonist group. Furthermore, the meta-analysis demonstrated that the combination of indobufen with clopidogrel therapy for a duration of 12 months was noninferior concerning the 1-year composite of MACCE when compared to the aspirin DAPT group. The relative risk was calculated at 1.58 with a 95% confidence interval of 0.72–3.38 (refer to Figure 2).

### 3.3. Primary Safety Endpoint

**Figure 3:**
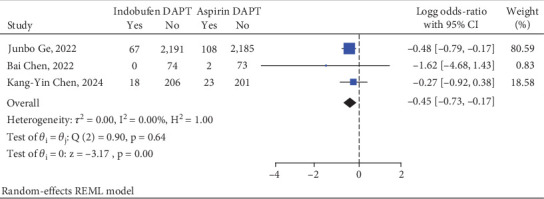
Indobufen-based DAPT versus aspirin-based DAPT as the primary safety endpoint.

The occurrence of BARC Types 2, 3, or 5 was significantly lower in the indobufen DAPT group when compared to the aspirin DAPT group, recorded at 3.33% in contrast to 5.13% (relative risk = 0.35, 95% confidence interval [0.18–0.67]) (Figure 3).

### 3.4. Gastrointestinal Reactions

**Figure 4:**
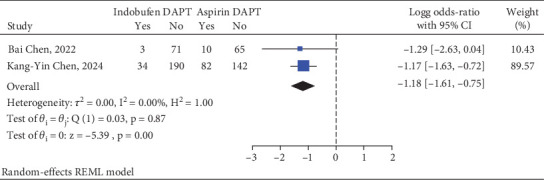
Indobufen-based DAPT versus aspirin-based DAPT on gastrointestinal reactions.

Gastrointestinal reactions that were clearly defined were documented in two RCTs. These reactions were observed in 37 patients (12.41%) from the indobufen DAPT cohort, as well as in 92 patients (30.77%) belonging to the aspirin DAPT cohort. Furthermore, a meta-analysis indicated that the incidence of gastrointestinal reactions was significantly lower in the indobufen DAPT group compared to the aspirin DAPT group, showing a relative risk of 0.06 and a 95% confidence interval of 0.03–0.18 (refer to Figure 4).

### 3.5. Potential Bias in Studies

We systematically excluded three studies, and the results exhibited no significant differences. The modifications made in this meta-analysis suggest a low sensitivity, resulting in findings that are more robust and credible (see Figure S1). We assessed publication bias through the creation of funnel plots and the application of Egger's test. The results from both the radial plot and the funnel plot suggest the presence of publication bias (refer to Figure S1). Given the limited research on indobufen, some heterogeneity issues in this article are unavoidable. This supplement (Table 1) shows heterogeneity across studies and supports using a random-effects approach.

## 4. Discussion

Numerous clinical studies have confirmed that after PCI or CABG, patients on DAPT have a significantly lower rate of ischemic events compared to those on aspirin monotherapy. In recent years, the advancement of PCI technology in China has been remarkable, with the annual volume of PCI procedures approaching 1 million and a corresponding increase in the number of patients receiving DAPT. Unlike conventional regimens, DAPT offers various new options for medication selection, administration timing, and optimal treatment duration. Particularly, a substantial proportion of patients undergoing PCI also present with atrial fibrillation (AF), necessitating the use of oral anticoagulants (OACs) to prevent ischemic stroke or systemic embolism. However, the concurrent use of OACs and DAPT significantly increases the risk of bleeding. Compared to triple antithrombotic therapy (TAT), dual antithrombotic therapy (DAT) has demonstrated a reduction in bleeding events but is associated with a higher incidence of stent thrombosis [20]. The optimization of antithrombotic and anticoagulation regimens presents considerable challenges. Analyzing diverse DAPT regimens can provide valuable insights for clinical practice, thereby enhancing the clinical net benefit while minimizing ischemia-related events. This, in turn, contributes to improved secondary prevention in patients with coronary artery disease and lessens both individual and societal health burdens.

Aspirin is fundamental in treating CAD and is classified as a Class I agent by both national and international clinical guidelines. Various studies indicate that “aspirin resistance” affects 5%–60% of individuals on aspirin therapy, leading to a higher rate of thrombotic recurrence events [21]. Indobufen, a platelet COX inhibitor created in Italy in 1970 and initially approved there in August 1984, is the sole reversible selective multitarget antithrombotic drug. It reversibly inhibits COX-1, exhibits low prostaglandin inhibition, leads to minimal gastrointestinal effects, and carries a low risk of hemorrhagic complications [2]. Prior studies have shown that indobufen inhibits platelet aggregation like aspirin but is more tolerable in the gastrointestinal tract [15]. Moreover, the antiplatelet effect of indobufen decreases more quickly, leading to a relatively low risk of bleeding. Thus, indobufen could serve as an alternative treatment for patients who have an aspirin allergy or intolerance after stent placement. The existing studies have specifically examined the safety and efficacy of aspirin and indobufen as standalone treatments.

The meta-analysis reviewed in our study, which encompassed 12 RCTs, highlights that indobufen presents specific advantages in comparison to other anticoagulant treatments [12]. However, in the subgroup analysis conducted in this study, an increased risk of stroke was noted in the indobufen cohort. Yet this finding may stem from inherent flaws in the study's design. In the two-stroke studies involving Chinese populations, the intervention in the INSURE experiment used indobufen versus aspirin [22], whereas the Liu et al. [23] study employed combinations of clopidogrel with indobufen versus clopidogrel with aspirin. Conducting a meta-analysis between these two groups disrupts the balance and comparability of the study design, a concern frequently observed in several RCTs focused on cardiovascular disease that were part of this review.

Thus, whether the dosing strategies identified in these studies apply to DAPT is still uncertain. Additionally, the effectiveness of indobufen used in conjunction with a P2Y12 receptor inhibitor compared to aspirin with a P2Y12 receptor inhibitor in clinical trials remains unclear, creating more uncertainties. Consequently, this meta-analysis aims to evaluate indobufen-based DAPT's extended safety and efficacy by reviewing relevant RCTs and previous meta-analyses that incorporate indobufen in conjunction with P2Y12 receptor inhibitors for clinical application.

Bai et al.'s [18] study findings indicate that the combination of indobufen and clopidogrel provides a more effective inhibition of platelet function than clopidogrel alone. This combined therapy could be an advantageous choice for antiplatelet treatment postcoronary stent implantation in patients experiencing ACSs, particularly those with severe hypersensitivity responses to aspirin resistance. Dai et al.'s findings [19] show that indobufen's antiplatelet effect is similar to aspirin's after PCI in patients with stable coronary artery disease. Indobufen may represent a viable option for patients exhibiting severe allergic reactions, resistance, or intolerance to aspirin subsequent to coronary stenting. Nonetheless, this conclusion necessitates further multicenter studies to rigorously evaluate the efficacy and safety of this novel antiplatelet agent in individuals with coronary artery disease. Bai et al. [18] revealed that the efficacy of indobufen in combination with clopidogrel was essentially comparable to that of aspirin in combination with clopidogrel in terms of antiplatelet therapy regimens for the prevention of restenosis of the bridge vessel 1 year after CABG, and the occurrence of gastrointestinal side effects associated with indobufen was significantly lower, which makes it worthwhile to conduct further studies to explore its potential as an option for antiplatelet therapy after CABG in the clinical setting. Previous studies have shown that the efficacy of indobufen in combination with P2Y12 receptor inhibitor antiplatelet therapy is comparable to that of aspirin in combination with P2Y12 receptor inhibitor antiplatelet therapy, and analyses of the safety and efficacy of indobufen as a single agent have further advanced its validation in clinical trials. The OPTION [20] study revealed that in Chinese patients exhibiting negative cardiac troponin levels who underwent drug-eluting stent implantation, the implementation of indobufen-based DAPT significantly diminished the risk of adverse net clinical outcomes over a 1-year period in comparison to aspirin-based DAPT. The noted reduction is mainly attributed to fewer hemorrhagic events and no significant rise in ischemic events. This research is notably the first major RCT aimed at evaluating the effectiveness and safety of replacing aspirin with a P2Y12 receptor inhibitor in patients who are receiving PCIs with drug-eluting stent implantation. For the first time, the effectiveness and safety of substituting aspirin with a P2Y12 receptor inhibitor have been thoroughly assessed among a large patient population in these interventions. Our analysis concludes that using indobufen alongside a P2Y12 inhibitor is more effective than the standard combination of aspirin and a P2Y12 receptor inhibitor, especially in terms of safety by reducing hemorrhagic events while keeping ischemic incident rates stable. This finding is consistent with previous studies, reinforcing the notion that indobufen remains a viable alternative to aspirin in the domain of DAPT. Furthermore, indobufen continues to be extensively utilized among patients exhibiting aspirin intolerance in East Asia; however, it is noteworthy that the majority of patients with ACS and aspirin intolerance were excluded from the OPTION trial. Retrospective analysis from the ASPIRATION registry revealed that indobufen had the same risk of MACCE but a lower risk of bleeding after PCI than aspirin from a real-world perspective [24]. The OPTION study [17], Bai et al.'s study [18], and the ASPIRATION registry [24] focused only on the safety and effectiveness of indobufen in CCS, and there is still a lack of evidence to recommend the use of indobufen in ACS. Studies are still warranted.

To assess the effectiveness of indobufen-based DAPT, particularly in patients with ACS, Pan et al.'s study [22] demonstrated that indobufen's efficacy and safety are comparable to those of aspirin in Chinese patients with AMI after PCI. Since then, indobufen's safety and effectiveness in both acute and chronic myocardial ischemia syndromes have been thoroughly evaluated and verified.

The three included clinical trials, respectively, demonstrated the efficacy and safety of indobufen in various myocardial ischemic events. Our meta-analysis further corroborated the long-term safety of indobufen; however, its efficacy remains questionable. In the defined ischemic events, the relative risk was 1.58 (95% CI, 0.72–3.38). While a statistical difference was observed between the groups, the upper limit of the 95% CI for the RR significantly exceeded 1, indicating that the long-term use of indobufen for the prevention of ischemic events is constrained. Furthermore, given the elevated cost of indobufen, conducting an economic cost-utility analysis represents a significant issue that warrants careful consideration.

Certainly, the present analysis primarily reflects DAPT based on clopidogrel, particularly within the Chinese cohort, which may restrict its applicability to the broader ACS population. Greater emphasis should be directed toward clinical trials involving DAPT regimens utilizing ticagrelor and prasugrel, as these align more closely with current standards of treatment for ACS. It is noteworthy that studies such as the TICO [25] and TWILIGHT [26] trials have demonstrated the safety and efficacy of ticagrelor monotherapy following short-term DAPT, even in complex PCI settings. These findings support the progressive paradigm of customizing antithrombotic strategies beyond the foundational clopidogrel regimen. Based on population, regimen, dosing, and drug heterogeneity, we emphasize that future studies should aim to stratify risk and evaluate antithrombotic strategies within more homogeneous populations to refine clinical applicability.

In summary, this study strongly recommends reassessing the optimal use of indobufen and encourages exploring alternative strategies to improve patient safety and treatment outcomes. While lowering bleeding risks without increasing ischemia risk, previous meta-analyses show that in patients undergoing complex PCI, P2Y12 inhibitor monotherapy offers greater safety regarding major bleeding events and does not raise the risk of ischemic incidents compared to standard DAPT. It also seems to decrease the risk of myocardial infarction, though this needs confirmation through focused clinical trials [13]. In our study, all indolebufenn DAPTs were combined with clopidogrel, rather than ticagrelor or prasugrel. Additionally, P2Y12 inhibitors were not used alone as a control for revascularization. These are all directions that need to be re-evaluated in the future.

There are several limitations: (1) Some studies from China were excluded because they did not describe the method of randomization and allocation concealment, there was selection bias, and there was implementation bias. The decision to include these analyses aimed to ensure transparency and reliability but did not give a full view of potential biases. Although inconclusive, the results offer tentative insights for future research. (2) Some of the included studies had a short follow-up period, and their long-term efficacy and safety could not be evaluated. (3) Only Chinese or English publications were included, and there may be some publication bias. (4) Currently, indobufen is primarily utilized within the Chinese market. Consequently, guidance on its application experience for other demographic groups should be approached with caution.

## 5. Conclusions

The use of DAPT with indobufen as an adjunct therapy presents an essential safety alternative to traditional antithrombotic agents; however, its efficacy in reducing the risk of ischemia may not be comparable. This underscores the need for re-evaluating the optimal application of indobufen and fosters the exploration of alternative strategies to improve patient safety and therapeutic outcomes. The assessment of indobufen in conjunction with P2Y12 DAPT within a Chinese population undergoing revascularization constitutes the foundation of this study.

## Figures and Tables

**Table 1:** Characteristics of the included studies.

**Study**	**Year**	**Country**	**Sample size**	**Age**	**Follow-up time**	**Treatment**	**Phenotye**
Junbo Ge, [17]	2022	China	4551	61.1 ± 8.3	12 m	I 0.1 bid + C 75 qd vs. A 0.1 qd + C 75 qd	Patients with negative cardiac troponin undergoing DES implantation
Bai et al., [18]	2022	China	152	60.3 ± 6.6	12 m	I 0.1 qd + C 75 qd vs. A 0.1 qd + C 75 qd	Bridge vessels at 1 year after off-pump coronary artery bypass grafting
Kang-Yin Chen, [19]	2024	China	1451	72.0 ± 11.0	12 m	I 0.1 qd + C 75 qd vs. A 0.1 qd + C 75 qd	Acute myocardial infarction

Abbreviations: A: aspirin; ACS: acute coronary syndrome; C: clopidogrel; DES: coronary drug-eluting stent; I: indobufen; PCI: percutaneous coronary intervention.

## Data Availability

Data sharing is not applicable to this article as no new data were created or analyzed in this study.

## Nomenclature

CAD: coronary atherosclerotic heart disease
PCI: percutaneous coronary intervention
CABG: coronary artery bypass grafting
COX: cyclooxygenase
NSAIDs: nonsteroidal anti-inflammatory drugs
DAPT: dual antiplatelet therapy
MACCE: major adverse cardiovascular and cerebrovascular events
BARC: bleeding academic research consortium
AF: atrial fibrillation
OAC: oral anticoagulants
DAT: dual antithrombotic therapy
RCT: randomized controlled trial
DES: drug-eluting stent
ACS: acute coronary syndrome
CCS: chronic coronary syndrome

## Supporting Information

Supporting Information Additional supporting information can be found online in the Supporting Information section. Figure S1: Heterogeneity and publication bias analysis. (A) Risk of bias and applicability concerns. (B) Sensitivity analysis. (C) Funnel plots. (D) Galbraith plots. Table S1: The results of the heterogeneity analysis of the meta-analysis. Figure S2: An alternative version of the PRISMA 2020 flow diagram for newly conducted systematic reviews, which incorporates searches of databases, registers, and other sources. An alternative version of the PRISMA 2020 flow diagram for newly conducted systematic reviews, which incorporates searches of databases, registers, and other sources (refer to Figure S2).
