# Supplementary material for: The Extended Safety and Efficacy of Indobufen in Conjunction With P2Y12 Receptor Inhibitors Among Patients Undergoing Revascularization: A Meta-Analysis and Overview
Source: Cardiovasc Ther. 2025 Nov 24;2025:5374818. doi: 10.1155/cdr/5374818 (PMC12668860; doi:10.1155/cdr/5374818)
Supplement: Supporting Information — Additional supporting information can be found online in the Supporting Information section. Figure S1: Heterogeneity and publication bias analysis. (A) Risk of bias and applicability concerns. (B) Sensitivity analysis. (C) Funnel plots. (D) Galbraith plots. Table S1: The results of the heterogeneity analysis of the meta-analysis. Figure S2: An alternative version of the PRISMA 2020 flow diagram for newly conducted systematic reviews, which incorporates searches of databases, registers, and other sources. An alternative version of the PRISMA 2020 flow diagram for newly conducted systematic reviews, which incorporates searches of databases, registers, and other sources (refer to Figure S2). [file 5374818.f1.zip › Supplement figure 2 PRISMA_2020_flow_diagram_new_SRs_v1.docx]

**Identification of studies via databases and registers**

Records removed *before the screening*:

Duplicate records removed (n = 307)

Records marked as ineligible by automation tools (n =45)

Records removed for other reasons (n = 65)

Records identified from:

Databases (n = 4)

Pubmed = 185

Web of Science = 309

EMBASE = 402

Cochrane = 108

**Identification**

Records screened

(n = 587)

Records excluded

(n =280)

Reports sought for retrieval

(n = 307)

Reports not retrieved

(n = 68)

**Screening**

Reports excluded:

No full ethical report (n = 90)

Not indobufen DAPT(n=15)

Ongoing RCT (n = 10)

Not RCT, a single-arm observational trial (n = 10)

No sufficient data (n = 14)

A retrospective case-control study (n = 75)

Case series analysis (n = 18)

Health volunteers (n = 3)

Reports assessed for eligibility

(n =239)

Studies included in review

(n = 3)

**Included**

1. No complete ethical report, 90; 2. No Dual Antiplatelet Therapy (DAPT) or duration < 6 months,15 ;3. No indobufen DAPT, 1;4. Enrolled in randomized controlled trial (RCT), 10; 5. Single-arm observational trial, 10; 6. Insufficient data, 14; 7. Retrospective case-control study, 75; 8. Analysis from case series, 18; 9. Healthy volunteers, 3
