# Supplementary figures and images for: The Extended Safety and Efficacy of Indobufen in Conjunction With P2Y12 Receptor Inhibitors Among Patients Undergoing Revascularization: A Meta-Analysis and Overview
Source: Cardiovasc Ther. 2025 Nov 24;2025:5374818. doi: 10.1155/cdr/5374818 (PMC12668860; doi:10.1155/cdr/5374818)

A

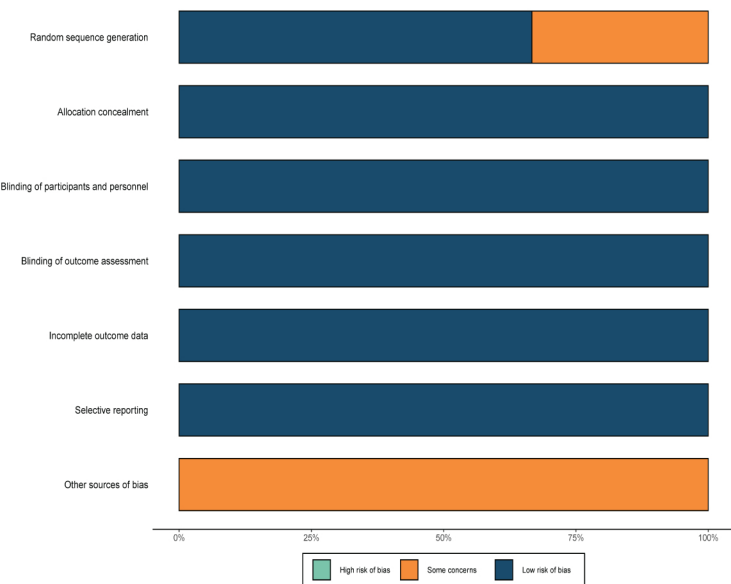

B

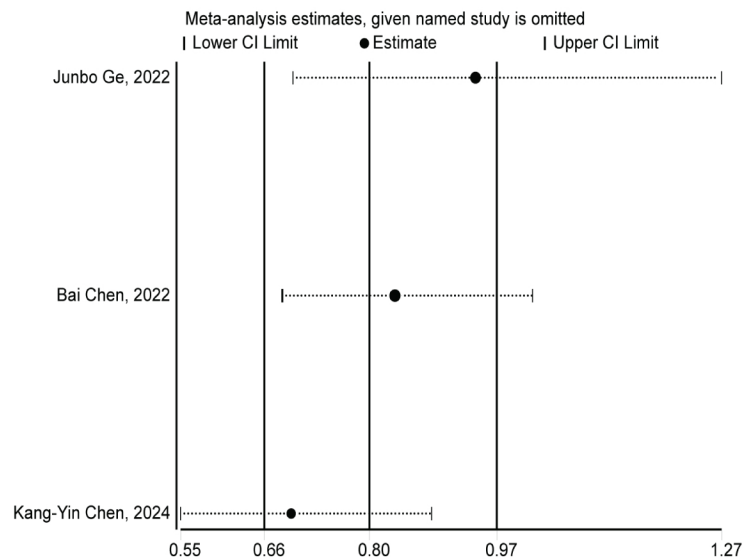

C

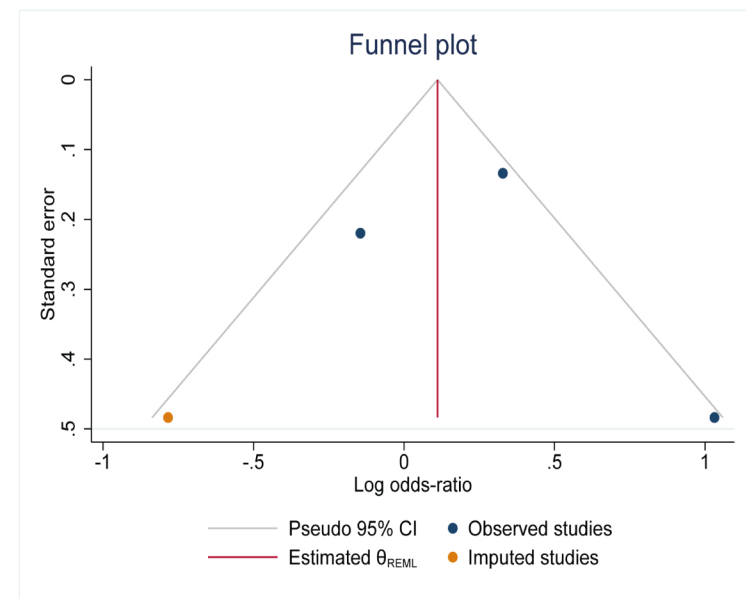

D

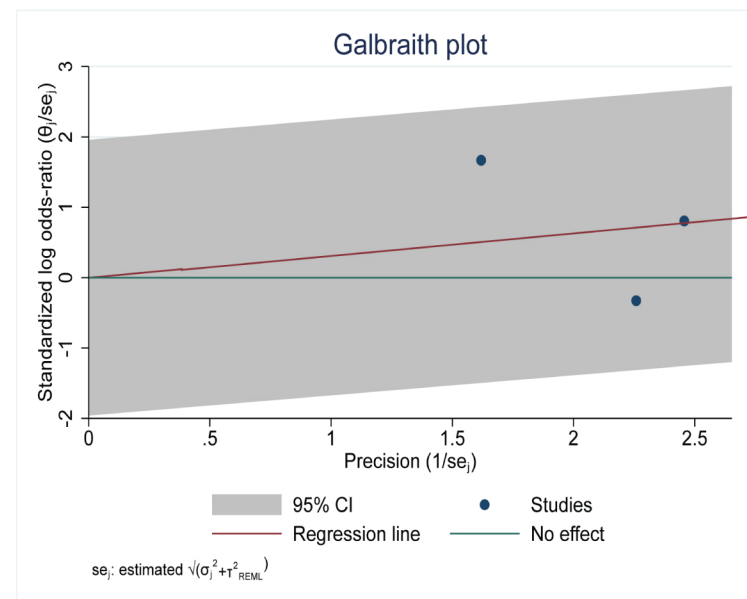

Supplement: Supporting Information — Additional supporting information can be found online in the Supporting Information section. Figure S1: Heterogeneity and publication bias analysis. (A) Risk of bias and applicability concerns. (B) Sensitivity analysis. (C) Funnel plots. (D) Galbraith plots. Table S1: The results of the heterogeneity analysis of the meta-analysis. Figure S2: An alternative version of the PRISMA 2020 flow diagram for newly conducted systematic reviews, which incorporates searches of databases, registers, and other sources. An alternative version of the PRISMA 2020 flow diagram for newly conducted systematic reviews, which incorporates searches of databases, registers, and other sources (refer to Figure S2). [file 5374818.f1.zip › supplementary Figure 1.pdf]
